# Association between ambient temperature and lower urinary tract symptoms: a community-based survey

**DOI:** 10.1590/S1677-5538.IBJU.2015.0159

**Published:** 2016

**Authors:** Sung Ryul Shim, Jae Heon Kim, Jong Ho Won, Eun Seop Song, Yun Seob Song

**Affiliations:** 1 Institute for clinical molecular biology research, Soonchunhyang University Hospital, Soonchunhyang University School of Medicine, Seoul, Korea;; 2 Department of Urology, Soonchunhyang University Hospital, Soonchunhyang University School of Medicine, Seoul. Korea;; 3 Department of Internal Medicine, Soonchunhyang University School of Medicine, Seoul, Korea;; 4 Department of Obstetrics and Gynecology, Inha University School of Medicine, Incheon, Korea

**Keywords:** Lower Urinary Tract Symptoms, Prostatic Hyperplasia, Temperature

## Abstract

**Purpose:**

The aim of this study was to evaluate the individual change of International prostate Symptom Score (IPSS) and Overactive Bladder Symptom Score (OABSS) in each patient by temperature conditions.

**Materials and Methods:**

The severity of lower urinary tract symptoms (LUTS) was explored using the IPSS and OABSS questionnaires that were completed by 2.486 subjects (923 males and 1.563 females) aged 60 years and older. Korea Meteorological Administration data was used to determine daily average temperature and daily temperature difference on the interview dates at each site.

**Results:**

The mean IPSS and mean age for males was 13.45±8.24 and 75.03±6.20 years, respectively. The mean OABSS and mean age for females was 4.41±3.10 and 73.74±6.03years, respectively. Daily average temperature and daily temperature difference ranged from-3.4-28.3^o^C and 2.2-16.9^o^C, respectively. Age was a significantly risk factor for IPSS, OABSS, and QoL (P<0.001, <0.001, and 0.005, respectively). After multiple regression analysis, daily average temperatures did not show a statistically significant change in IPSS and OABSS. Only daily temperature differences were associated with male LUTS.

**Conclusions:**

While LUTS could be worsened in low temperatures generally, IPSS and OABSS were not affected by daily average temperature conditions. Daily temperature differences may be more influential than daily average temperatures.

## INTRODUCTION

**Figure 1:**
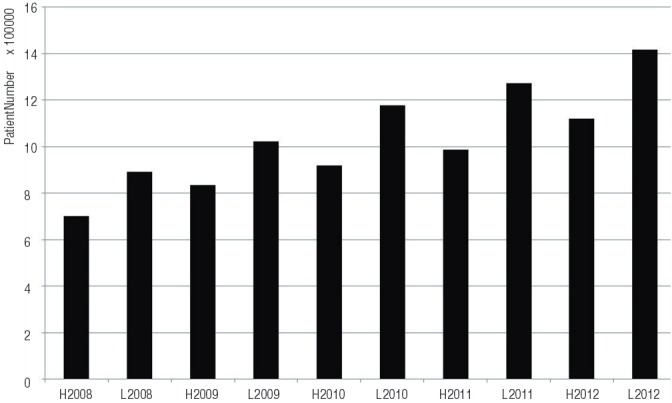
Seasonal benign prostatic hyperplasia (international classification of disease code; N40) patient number from 2008 to 2012. L, low daily average temperature (<10ºC), (Jan. to Mar., Nov., Dec.); H, high daily average temperature (>20ºC) (Jun. to Sept.). The data set of National Health Insurance in Korea.

Variance in environmental temperature has been associated with various diseases. The documented more frequent occurrence of myocardial infarction in cold temperatures is attributed to increase in plasma viscosity, serum cholesterol levels, blood pressure, sympathetic nervous activities, and platelet aggregation (1, 2). Concerning the association between benign prostate hyperplasia (BPH)/lower urinary tract symptoms (LUTS), according to the BPH/LUTS patients-based dataset of five-years (2008-2012) of National Health Insurance in Korea, seasonal variations of visiting hospital patients between summer (June to September) and winter (January to March, November, December), it was highest in 2011 (29.0%) and lowest in 2009 (22.6%) (Figure-1).

Accessed November 28 2014.

Seasonal variations of LUTS were reported in several small longitudinal studies; cold environmental stress and ambient temperature change elicited urinary sensations and frequent urination along with increasing heart rate and blood pressure (3, 4). However, seasonal changes were not observed in the International Prostate Symptom Score (IPSS), storage symptom score, voiding symptom score, and quality of life (QoL) (5, 6). As well, changes in fluid temperature did not significantly change the threshold volume of bladder sensation or increase the incidence of idiopathic detrusor overactivity in urodynamic studies (7). There is no general consensus about the effect of environmental temperature on LUTS associated with BPH and overactive bladder (OAB). Therefore, we focused on ambient temperature as an environmental factor affecting LUTS associated with BPH or OAB, and attempted to explain the temperature difference changes in BPH or OAB severity.

To date, no large cross-sectional surveys have been performed to investigate the association between daily temperature and LUTS. The aim of this study was to investigate this association by community-based survey.

## MATERIALS AND METHODS

### Methodology

**Figure 2:**
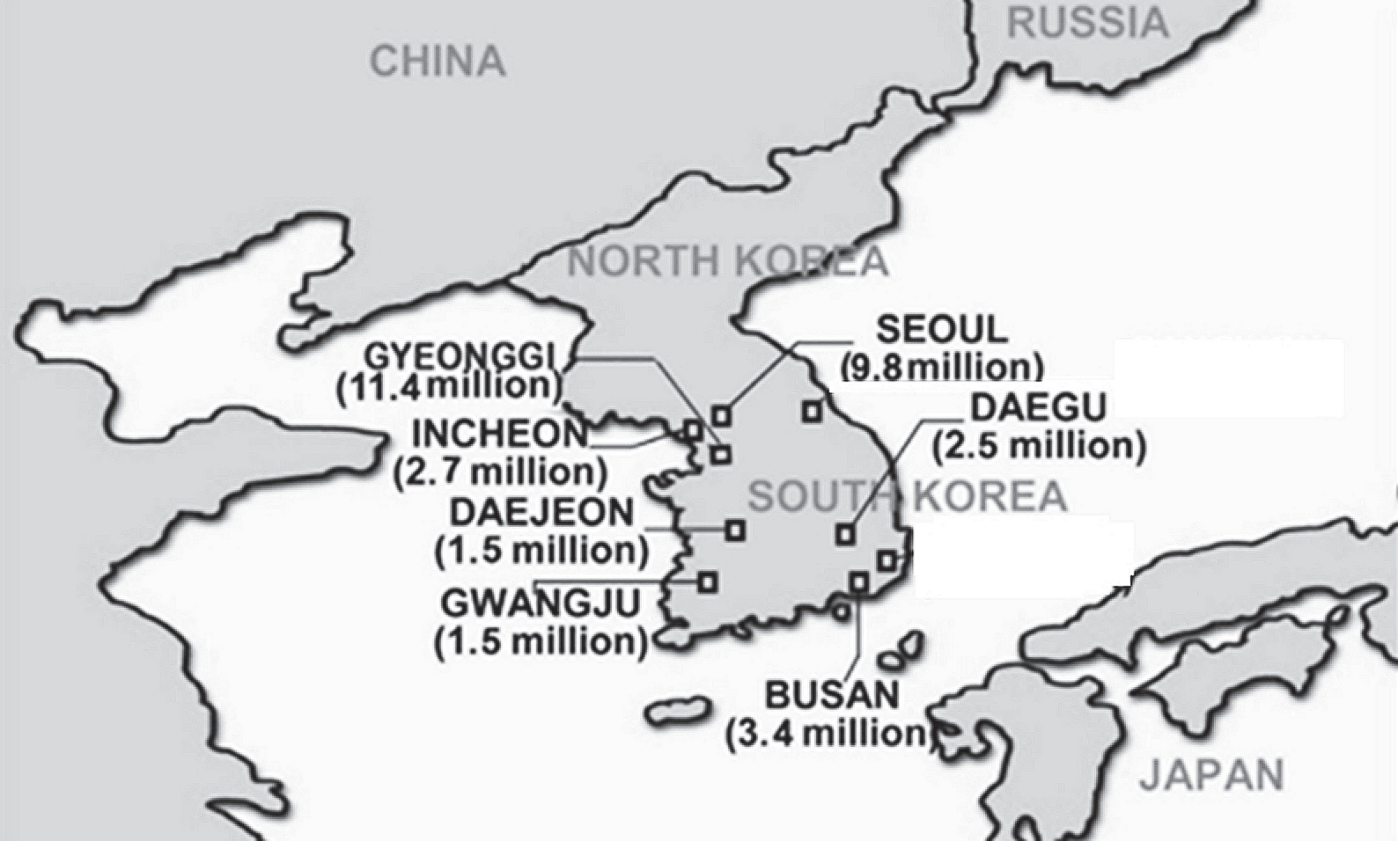
Location of seven major provinces in this study. The numbers in parentheses refer to population.

This was a cross-sectional study. One investigator conducted face-to-face interviews with all study participants at senior welfare centers in South Korea between August 2010 and November 2012 using the International prostate Symptom Score (IPSS) and Overactive Bladder Symptom Score (OABSS) questionnaires. The survey was conducted 36 times in 34 cities within seven major areas of South Korea: Seoul, Gyeonggi, Incheon, Daejeon, Daegu, Gwangju, and Busan (Figure-2).

### Study Area

South Korea is located in the southern portion of the Korean Peninsula, which extends about 1.100km (680mi) from the Asian mainland. The mountainous peninsula is flanked by the Yellow Sea to the west and East Sea to the east. The country, including all its islands, lies between latitudes 33º and 39º N, and longitudes 124º and 130ºE. Its total area is 100.188 square kilometers (http://www.ngii.go.kr/kor/board/view.do?rbsIdx=103&idx=66). South Korea tends to have a humid continental climate and a humid subtropical climate, and is affected by the East Asian monsoon. South Korea has four distinct seasons: spring, summer, autumn, and winter. Winters can be extremely cold with the minimum temperature dropping below -20ºC (-4ºF) in the inland region of the country: in Seoul, the average January temperature range is -7 to 1ºC (19 to 34ºF), and the average August temperature range is 22 to 30ºC (72 to 86ºF). Summer can be uncomfortably hot and humid, with temperatures exceeding 30ºC (86ºF) in most parts of the country (http://countrystudies.us/south-korea/31.htm).

### Study population

The study used data from a community-based interview survey conducted with 2.486 male (n=923) and female (n=1.563) subjects 60 years of age and older who provided voluntary consent to participate in the questionnaire survey. To enhance the validity of research, exclusion criteria were prior urological surgery; prior treatment for BPH, prostate cancer or OAB; evidence of a neurological condition; history of a malignancy; evidence of urinary tract infection; evidence of psychiatric illness; and evidence of alcohol or substance abuse.

### Temperature data collection

In South Korea, the Korea Meteorological Administration maintains a meteorological observational network of 94 stations that measure daily average temperature, daily temperature difference, daily maximum temperature, daily minimum temperature, and daily amount of precipitation (http://www.kma.go.kr). This allowed retrieval of data concerning daily average temperature and daily temperature difference on the interview dates at the specific measurement sites.

## QUESTIONNAIRES

IPSS Questionnaire: The severity of LUTS associated with BPH for males was measured by use of the IPSS questionnaire, which is based on the American Urological Association symptom index, with one additional question regarding QoL. The Korean version of the IPSS was verified in terms of its relevance and reliability, and it is now the most typical diagnostic instrument for LUTS in Korea.

The questionnaire consists of eight items, which include seven 6-point scale questions about symptoms of residual urine sensation, urinary frequency, interrupted stream, urinary urgency, weak urinary stream, urinary hesitancy, and nocturia, and one 7-point scale question on patient satisfaction with their urinary condition. Based on the previously defined criteria (8), symptom severity was divided into three groups: mild (symptom score 0-7), moderate (8-19), and severe (20-35). QoL in LUTS patients or their level of satisfaction was represented by seven grades: “no problem” (0 point=very satisfied), “all right” (1 point), “somewhat satisfied” (2 points), “half-satisfied, half-dissatisfied” (3 points), “somewhat dissatisfied” (4 points), “distressed” (5 points), and “can’t stand it” (6 points=very dissatisfied).

### OABSS Questionnaire

The severity of LUTS associated with OAB for females was measured by use of the OABSS questionnaire. The OABSS was developed and validated in Japanese populations (9). The OABSS comprises only four questions regarding daytime frequency, nocturia, urgency, and urgency incontinence, and evaluates relevant symptoms from the viewpoint of the patient. Performance of the OABSS is simple and quick, and a good agreement between OABSS items and the corresponding diary variables was found in a clinical trial with anticholinergics (10). The Korean version of the OABSS was verified in terms of its relevance and reliability.

### Age

Age is an important factor that has an impact on generation-specific prevalence of BPH and OAB. Therefore, this study queried each participant’s date of birth.

### Classification of temperature and statistical analyses

Classification of temperature was based on the daily average temperature (low, <10^o^C; medium, 10-20^o^C; high>20^o^C) as previously described (6) and daily temperature difference in tertiles (low, <8^o^C; medium 8-10^o^C; high>10^o^C). To examine the influence of topographical characteristics, the population was divided into two groups: coastal area (Incheon, Busan) and inland area (Seoul, Gyeonggi, Daejeon, Daegu, Gwangju). To examine the relationship between BPH/OAB and age in the population, an analysis of variance (11) and a post hoc analysis were performed to identify any differences in IPSS/OABSS among each age group. Multiple linear regression analysis with IPSS/OABSS as the response variable, and daily average temperature, daily temperature difference, and age as explanatory variables was done. All data are presented as mean and standard deviation (SD). Statistical analysis was performed using SPSS version 21.0 software (IBM, New York, NY, USA) and STATA version 11.2 software (StataCorp LP, Texas, USA). All statistics were two-tailed and P-values <0.05 were considered to be significant.

## RESULTS

The mean IPSS and mean age for the 923 males was 13.45±8.24 and 75.03±6.20 years, respectively. The mean OABSS and mean age for the 1.563 females was 4.41±3.10 and 73.74±6.03 years, respectively (Table-1). Daily average temperature ranged from-3.4^o^C to 28.3^o^C, with the daily temperature difference ranging from 2.2^o^C to 16.9o C (Table-2).

**Table 1:** Characteristics of participants and temperature.

	Male (n = 923)		Female (n = 1,563)	
Age	75.03±6.20			73.74±6.03
IPSS	13.45±8.24		OABSS	4.41±3.10
QOL	2.84±1.68			

Age groups				

60 - 69	172 (18.6)			392 (25.1)
70 - 79	539 (58.4)			911 (58.3)
Over 80	212 (23.0)			260 (16.6)

IPSS severity groups			OABSS severity groups	

Mild	284 (30.8)			1,117 (71.5)
Moderate	454 (49.2)			378 (24.2)
Severe	185 (20.0)			68 (4.4)

Daily average temperature groups				

Low	267 (28.9)			452 (28.9)
Medium	296 (32.1)			590 (37.7)
High	360 (39.0)			521 (33.3)

Daily temperature difference groups				

Low		349 (37.8)		475 (30.4)
Medium		226 (24.5)		519 (33.2)
High		348 (37.7)		569 (36.4)

Figures are means ± SD or numbers with percentages in parentheses.

IPSS, international prostate symptom score. OABSS, overactive bladder symptom score. QOL, quality of life.

IPSS severity groups – mild group (≤7 of IPSS), moderate group (8 - 19 of IPSS), severe group (≥20 of IPSS). OABSS severity groups – mild group (≤ 5 of OABSS), moderate group (6 - 11 of OABSS), severe group (≥ 12 of OABSS). Daily average temperature groups – low (<10.0^0^C), medium (10.0 - 20.0^0^C), high (>20.0^0^C). Daily temperature difference groups - low (<8.0^0^C), medium (8.0 - 10.0^0^C), high (>10.0^0^C).

**Table 2:** Daily average temperature and daily temperature difference by interview date at measurement sites.

Male (n = 923)						Female (n = 1,563)					

DAT*(^o^C)	n	%	DTD†(^o^C)	n	%	DAT*(^o^C)	n	%	DTD†(^o^C)	n	%
-3.4	27	2.9	2.2	21	2.3	-3.4	22	1.4	2.2	31	2.0
-0.7	27	2.9	4.4	17	1.8	-0.7	55	3.5	4.4	15	1.0
2.2	22	2.4	6.0	29	3.1	2.2	46	2.9	6.0	36	2.3
2.6	27	2.9	6.2	23	2.5	2.6	25	1.6	6.2	43	2.8
3.7	17	1.8	6.5	59	6.4	3.7	15	1.0	6.5	70	4.5
4.8	22	2.4	7.0	64	6.9	4.8	61	3.9	7.0	91	5.8
5.4	36	3.9	7.3	24	2.6	5.4	33	2.1	7.3	12	0.8
6.0	25	2.7	7.5	25	2.7	6.0	63	4.0	7.5	37	2.4
7.9	39	4.2	7.6	25	2.7	7.9	71	4.5	7.6	63	4.0
9.5	4	0.4	7.8	34	3.7	9.5	30	1.9	7.8	42	2.7
9.6	21	2.3	8.0	28	3.0	9.6	31	2.0	8.0	35	2.2
10.2	4	0.4	8.1	3	0.3	10.2	71	4.5	8.1	48	3.1
10.8	34	3.7	8.2	4	0.4	10.8	42	2.7	8.2	30	1.9
11.4	14	1.5	8.5	18	2.0	11.4	40	2.6	8.5	108	6.9
11.6	13	1.4	8.5	22	2.4	11.6	29	1.9	8.5	61	3.9
13.0	63	6.8	8.9	42	4.6	13.0	94	6.0	8.9	77	4.9
13.3	61	6.6	9.0	42	4.6	13.3	89	5.7	9.0	43	2.8
15.1	53	5.7	9.3	6	0.7	15.1	28	1.8	9.3	38	2.4
15.4	3	0.3	9.5	22	2.4	15.4	34	2.2	9.5	46	2.9
18.0	3	0.3	9.9	67	7.3	18.0	48	3.1	9.9	68	4.4
19.1	42	4.6	10.0	98	10.6	19.1	77	4.9	10.0	8	0.5
19.4	6	0.7	10.4	75	8.1	19.4	38	2.4	10.4	104	6.7
20.2	22	2.4	10.6	4	0.4	20.2	35	2.2	10.6	71	4.5
20.5	19	2.1	10.7	27	2.9	20.5	13	0.8	10.7	103	6.6
22.2	24	2.6	11.0	13	1.4	22.2	12	0.8	10.7	25	1.6
24.2	23	2.5	11.5	30	3.3	24.2	43	2.8	11.0	29	1.9
25.4	123	13.3	11.6	19	2.1	25.4	45	2.9	11.5	89	5.7
25.7	29	3.1	14.4	21	2.3	25.7	36	2.3	11.6	13	0.8
25.8	28	3.0	16.9	61	6.6	25.8	35	2.2	14.4	38	2.4
26.1	42	4.6				26.1	43	2.8	16.9	89	5.7
26.3	32	3.5				26.3	48	3.1			
28.3	18	2.0				27.4	103	6.6			
						28.3	108	6.9			

* Daily average temperature † Daily temperature difference.

One-way ANOVA analysis was conducted to determine the age-related risk of BPH, OAB, and QoL. The risk of BPH significantly increased with age (12.65±7.96 for those aged 60-69 years and 15.21±8.86 for those over 80 years; P=0.002). The risk of OAB significantly increased with age (3.93±2.82 and 4.90±3.467 for the respective age groups; P<0.001). The risk of QoL increased significantly with age (2.83±1.75 and 3.21±1.53 for the respective age groups; P=0.001) (Table-3). To evaluate the influence of topographical characteristics, there was no difference of IPSS, QoL, and OABSS between coastal and inland area (Table-4). And also there was no statistical significance for identifying which individual item of IPSS and OABSS was related to the daily average temperature except for QoL item (Table-5).

**Table 3:** Age-specific severity of IPSS, OABSS and QoL.

	IPSS				OABSS				QoL			
			
Age group	n (923)	Mean	SD	*P*	n (1.563)	Mean	SD	P	n (923)	Mean	SD	*P*
60- 69	172	12.65*	7.959	0.002	392	3.93	2.820	0.000	172	2.83*	1.754	0.001
70 – 79	539	13.01*	7.981		911	4.47*	3.082		538	2.70*	1.688	
Over 80	212	15.21	8.860		260	4.90*	3.459		212	3.21	1.525	

**IPSS** = International prostate symptom score; **OABSS** = Overactive bladder symptom score; **QoL** = Quality of life; **IPSS & QoL** are for male and OABSS is for female. P-value, one-way analysis of variances; * Same letters indicate no statistical significance based on Duncan multiple comparison.

**Table 4:** Comparison of topographical groups of IPSS, OABSS and QoL.

	IPSS				OABSS				QoL			
			
Area	n (923)	Mean	SD	*P*	n (1,563)	Mean	SD	*P*	n (923)	Mean	SD	*P*
Coastal area	207	13.78	8.241	0.767	433	4.65	3.193	0.056	207	2.94	1.704	0.864
Inland area	716	13.35	8.238		1130	4.31	3.059		716	2.81	1.667	

**IPSS** = International prostate symptom score; **OABSS** = Overactive bladder symptom score; **QoL** = Quality of Life; **IPSS & QoL** are for male and OABSS is for female; **P-value** = Student t-test. Coastal area (Incheon, Busan). Inland area (Seoul, Gyeonggi, Daejeon, Daegu, Gwangju).

**Table 5:** Comparison of daily average temperature groups in each item of IPSS, OABSS and QoL.

		IPSS				QOL					OABSS					
			
	DAT* group	n (923)	Mean	SD	*P*	DAT* group	n (923)	Mean	SD	*P*		DAT* group	n (1,563)	Mean	SD	*P*
Incomplete emptying (IPSS 1)	L	267	1.88	1.737	0.652	L	267	2.65	1.709	0.001	Frequency (OABSS 1)	L	452	0.40	0.622	0.723
	M	296	1.75	1.591		M	295	2.70	1.614			M	590	0.41	0.621	
	H	360	1.84	1.622		H	360	3.09	1.673			H	521	0.43	0.595	
Frequency (IPSS 2)	L	267	1.86	1.617	0.501						Nocturia (OABSS 2)	L	452	1.94	0.898	0.774
	M	296	1.95	1.511								M	590	1.90	0.908	
	H	360	2.01	1.603								H	521	1.90	0.938	
Intermittency (IPSS 3)	L	267	1.83	1.739	0.925						Urgency (OABSS 3)	L	452	1.09	1.480	0.101
	M	295	1.78	1.655								M	590	1.28	1.572	
	H	360	1.82	1.576								H	521	1.24	1.505	
Urgency (IPSS 4)	L	267	1.49	1.600	0.708						Urgency incontinence (OABSS 4)	L	450	0.77	1.278	0.064
	M	296	1.53	1.638								M	590	0.96	1.407	
	H	360	1.60	1.678								H	521	0.85	1.265	
Weak stream (IPSS 5)	L	267	2.36	1.736	0.927											
	M	296	2.42	1.705												
	H	360	2.38	1.721												
Straining (IPSS 6)	L	267	1.50	1.616	0.209											
	M	296	1.59	1.634												
	H	360	1.73	1.691												
Nocturia (IPSS 7)	L	267	2.40	1.259	0.657											
	M	296	2.31	1.291												
	H	360	2.39	1.308												
Total symptom score	L	267	13.24	8.347	0.702						Total symptom score	L	452	4.19	2.960	0.175
	M	296	13.29	7.865								M	590	4.55	3.293	
	H	360	13.73	8.463								H	521	4.43	2.986	

Daily average temperature groups – L (<10.0^o^C), M (10.0 - 20.0^o^C), H (>20.0^o^C). IPSS, international prostate symptom score. OABSS, overactive bladder symptom score. QOL, quality of life. IPSS & QOL are for male and OABSS is for female. P-value, one-way analysis of variances.

Figure-3 displays values with 95% confidence intervals after adjustment of age concerning the relationship between temperature factors and IPSS/OABSS. There was a weak negative correlation between IPSS and daily temperature difference. To examine this correlation in more detail, a multiple linear regression analysis was performed to assess the change in IPSS, OABSS, and QoL during one year with respect to risk factors for BPH and OAB. In the analysis, independent variables included age, daily average temperature, and daily temperature difference, and dependent variables included IPSS, OABSS, and QoL. Age was a significantly risk factor for IPSS, OABSS, and QoL (P<0.001, <0.001, and 0.005, respectively). Regression analysis found that a one-degree Celsius increase in daily temperature difference decreased the IPSS by-0.216 points (P=0.02) and a one-degree Celsius increase in daily average temperature increased the QoL by 0.021 points (P=0.001). Daily average temperatures did not show a statistically significant change in IPSS and OABSS (Table-6). The variance inflation factor among the explanatory variables ranged from 1.005 (lowest) to 1.017 (highest) in IPSS and from 1.016 (lowest) to 1.032 (highest) in OABSS. Multicollinearity was not significantly observed.

**Table 6:** Multiple linear regression analysis of IPSS, OABSS and QoL.

	IPSS			OABSS			QOL		
			
	B*	SE	*P*	B*	SE	*P*	B*	SE	*P*
Age (years)	0.177	0.043	0.000	0.059	0.013	0.000	0.025	0.009	0.005
Daily average temperature (^o^C)	0.030	0.030	0.322	0.007	0.009	0.433	0.021	0.006	0.001
Daily temperature difference (^o^C)	-0.216	0.093	0.020	-0.034	0.029	0.246	-0.015	0.019	0.423

**IPSS** = international prostate symptom score. **OABSS** = Overactive bladder symptom score. **QOL** = quality of life; **IPSS & QOL are** for male and OABSS is for female. * Unstandardized coefficients.

## DISCUSSION

Temperature has been linked with myocardial infarction (2), ischemic heart disease (12), brain-blood vessel obstruction (13), and respiratory infection (14). For urinary voiding symptoms, it is necessary to consider how ambient temperature changes affect IPSS in medicated patients (4). However, in Japan seasonal changes were found not to be associated with IPSS (6). Also, the report of an average temperature odds ratio of chronic prostatitis-like symptoms of 0.99 (range 0.98 to 1.00) was indicative of only a weak clinical significance in Korea (5). In this present study, average temperature was not a risk factor for LUTS for adjusted age in multiple regression analysis (P=0.322). Among the variables, only QoL revealed significant association, which was only prominent among male populations. Moreover, the significant level was marginal, which could be interpreted as temporary phenomenon and needs more validation.

Concerning urinary storage symptoms, patients without neurological diseases have a heightened perception of cold in the bladder during the ice water test than patients with neurological diseases (15). Storage symptoms, frequency, urgency, and nocturia are considerably affected by seasonal changes (16). However, changes in the temperature of fluid did not significantly change the threshold volume of bladder sensation or increase the incidence of idiopathic detrusor overactivity in urodynamic studies (7). In the present study, the average temperature did not demonstrate a risk of LUTS for adjusted age in multiple regression analysis (P=0.433).

There are several reasons for the varying results. First, several previous studies reported that cold stress induces detrusor overactivity in conscious rats, a finding that occurred with a high temperature change between the treatment group and the control group (△24^o^C=room temperature 28oC-low temperature 4ºC) (15, 17, 18). However, in the present study, the maximum daily temperature difference was 16.9^o^C, which was lower than in previous studies. Also, in general, elderly people have shorter exposure times and well-controlled body temperatures in the winter season due to their typically limited physical activity, which could lead to a smaller exposure to extreme temperature changes.

Second, the same previous studies measured outcomes during a short exposure time (20-40 min) (15, 17-19). However, in general, the human body’s activity changes with the seasons and gradually adapts to the exposure temperature throughout the season. This phenomenon may account for our finding that the temperature effect on the risk of BPH/OAB may not affect urinary symptoms in the general population. For instance, in a cold stress-induced detrusor overactivity model, when skin temperature stabilized after 20 min of low temperature (4±2ºC) exposure and was maintained for the duration of exposure (19), demonstrated that a momentary cold stimulus can act as a trigger for the urinary responses. Also, the results of this model were associated with a sudden decrease in skin temperature (19). This present study also supports previous results that the time-dependent reductions of low temperature stimulated responses represent an adaptive response that is universal in normal healthy humans. Moreover, individual efforts to maintain warmth through the wearing of heavy clothes and heating the environment could diminish the trigger effect of low temperature on LUTS.

It has generally been thought that more LUTS patients are associated with BPH/OAB-related need for hospital examination with regard to their urinary symptoms in low temperature circumstances. However, the present results indicate that this does not mean that the urinary severity of patients in low temperature environments is higher than high temperature exposure.

The guideline by the Japanese Urological Association recommends one of the conservative treatments that males with LUTS avoid exposing the lower body to cold temperature (20). However, the American Urological Association and European Association of Urology have not provided high-quality and reliable evidence about the influence of ambient temperature on LUTS (21, 22). Only the American Urological Association guideline on the management of BPH recommended future study of life style interventions (21). The present data address this recommendation.

There are several limitations to this study. In view of the imprecision of some geographical data, coupled with the fact that we used spatially-derived ambient temperature as a surrogate for personal temperature, the risk estimates presented here are clearly misclassified. Thus, our results may underestimate the true risk of LUTS associated with exposure to temperature in this population. However, as in most epidemiological surveys, there will be some errors in exposure classification. In this study, which was performed as a large-scale research project that covered seven major areas of South Korea, the collected data were judged to be sufficiently homogeneous. As a result, the misclassification of this study would most likely be non-differential with regard to temperature status. This would tend to bias the regression parameter toward null. Second, this study does not include the detailed biological data of each population, which means we could not determine clinical BPH or OAB. This is mainly due to the nature of this cross-sectional survey.

Topographical characteristics were taken into consideration with regard to variations in LUTS severity among areas. Since coastal and inland communities are evenly distributed in interview surveys areas 36 times in 34 cities in seven major areas of South Korea, there is no difference of IPSS, QoL, and OABSS between coastal and inland area. Thus, the topographical difference is also thought to be unrelated.

Lastly, we could not describe the longitudinal data to consider potential seasonal variation effects. The variance of daily temperature could not substitute for seasonal variation. Hence, this type of cross sectional study has to be repeated by seasonal sequence.

## CONCLUSIONS

Our findings did not demonstrate an increased clinically significant risk of BPH or OAB severity in connection with daily average temperature. Only daily temperature differences were associated with male LUTS. Daily temperature differences may be more influential than daily average temperatures. A large prospective study set will be needed to validate this association in the future.
